# Association Between Acute Gastroenteritis and Continuous Use of Proton Pump Inhibitors During Winter Periods of Highest Circulation of Enteric Viruses

**DOI:** 10.1001/jamanetworkopen.2019.16205

**Published:** 2019-11-27

**Authors:** Ana-Maria Vilcu, Laure Sabatte, Thierry Blanchon, Cécile Souty, Milka Maravic, Magali Lemaitre, Olivier Steichen, Thomas Hanslik

**Affiliations:** 1 Sorbonne Université, INSERM, Institut Pierre Louis d’épidémiologie et de Santé Publique (IPLESP UMRS 1136), Paris, France; 2 Real World Insight, IQVIA, La Défense Cedex, France; 3 Service de Rhumatologie, Hôpital Lariboisière, Assistance Publique–Hôpitaux de Paris (APHP), Paris, France; 4 Sorbonne Université, INSERM, Université Paris 13, Laboratoire d’informatique Médicale et d’Ingénierie des Connaissances en e-santé, LIMICS, Paris, France; 5 Service de Médecine Interne, Hôpital Tenon, Assistance Publique–Hôpitaux de Paris (APHP), Paris, France; 6 UVSQ, UFR de Médecine, Université de Versailles Saint-Quentin-en-Yvelines, Versailles, France; 7 Service de Médecine Interne, Hôpital Ambroise Paré, Assistance Publique–Hôpitaux de Paris (APHP), Boulogne Billancourt, France

## Abstract

**Importance:**

An increased risk of acute bacterial enteric infections has been reported among patients receiving proton pump inhibitor (PPI) therapy. The risk of acute gastroenteritis (AGE) of viral origin associated with continuous PPI exposure has been less studied.

**Objective:**

To investigate the association between continuous PPI therapy and AGE occurrence during winter epidemic periods when the circulation of enteric viruses is the highest.

**Design, Setting, and Participants:**

A matched cohort study was performed using a prospectively collected drug dispensing database from a large panel of community pharmacies in continental France. All patients recorded in the database during the 2015 to 2016 winter season, with documented age, sex, and use of an identifiable regular panel pharmacy, were eligible for the study. Each patient exposed to continuous PPI therapy was matched to 3 unexposed patients, according to year of birth, sex, and identifiable regular panel pharmacy. Analyses were performed between January 2017 and December 2018.

**Exposure:**

Continuous PPI use during the 2015 to 2016 AGE winter epidemic.

**Main Outcomes and Measures:**

The occurrence of at least 1 AGE episode during the 2015 to 2016 AGE winter epidemic was the main outcome. Episodes of AGE were identified using a previously validated algorithm based on drug dispensing data. Relative risks of AGE were estimated using a multivariable log-binomial model adjusted for age, sex, and treatments for chronic conditions.

**Results:**

There were 233 596 patients receiving PPI therapy (median [interquartile range] age, 71 [62-81] years; 55.8% female) and 626 887 matched patients not receiving PPI therapy (median [interquartile range] age, 70 [61-80] years; 56.3% female) included in the analyses. At least 1 AGE epidemic episode was identified in 3131 patients (1.3%) receiving PPI therapy and in 4327 patients (0.7%) not receiving PPI therapy. The adjusted relative risk of AGE for those receiving PPI therapy was 1.81 (95% CI, 1.72-1.90) for all ages considered, 1.66 (95% CI, 1.54-1.80) among those aged 45 to 64 years, 2.19 (95% CI, 1.98-2.42) among those aged 65 to 74 years, and 1.98 (95% CI, 1.82-2.15) among those aged 75 years and older.

**Conclusions and Relevance:**

Continuous PPI therapy was associated with an increased risk of developing AGE during periods of highest circulation of enteric viruses. These findings support the hypothesis that PPI use is associated with an increased risk of enteric viral infections.

## Key Points

**Question:**

Is continuous use of proton pump inhibitors (PPIs) associated with an increased risk of acute gastroenteritis during periods of highest circulation of enteric viruses?

**Findings:**

In this matched cohort study comparing 233 596 patients receiving continuous PPI therapy with 626 887 patients not receiving PPI therapy, the adjusted relative risk of occurrence of acute gastroenteritis during periods of highest circulation of enteric viruses was 1.81 times higher in patients receiving continuous PPI therapy than in those not receiving PPI therapy.

**Meaning:**

Continuous PPI use may be associated with an increased risk of enteric viral infections.

## Teaser

This cohort study investigates the association between continuous proton pump inhibitor therapy and acute gastroenteritis due to infection with enteric viruses during the 2015 to 2016 winter epidemic in France.

## Introduction

Proton pump inhibitors (PPIs) are drugs widely prescribed to reduce gastric acidity. Although they are considered globally safe, observational studies^1,2,3^ have reported significant adverse effects associated with their long-term use, such as osteoporotic-related fractures, vitamin B_12_ deficiency, kidney disease, or infections, particularly pulmonary and digestive tract infections. Numerous studies^4,5,6,7,8^ have reported an association between PPI use and bacterial enteric infections, such as isolated and recurrent *Clostridium difficile* infections. By reducing the secretion of hydrochloric acid produced by the stomach, PPIs may promote the growth of the gastrointestinal microflora, increase bacterial translocation, affect the gastrointestinal microbiome, and weaken the immune system.^9^

The association between continuous PPI use and acute gastroenteritis (AGE) caused by enteric viruses has been less studied. To our knowledge, only 1 study^10,11^ has investigated the association between PPI use and norovirus infections, but it was based on a small sample of inpatients and did not distinguish between continuous and short-term PPI therapy.

In the temperate northern hemisphere, peaks of AGE activity are observed every winter. They are mainly caused by infections with enteric viruses.^12,13,14^ This study aimed to investigate the association between continuous PPI exposure and the occurrence of AGE during winter epidemics when the circulation of enteric viruses is the highest, using community pharmacy drug dispensing data issued for ambulatory care prescriptions, collected in a large French nationwide database during the 2015 to 2016 winter season.

## Methods

### Study Design and Data Sources

Considering the association between PPI prescriptions and patient age and the large volume of data available, a matched retrospective cohort design using prospectively collected ambulatory care–prescribed drug dispensing data from community pharmacies was considered appropriate to investigate this association.^15,16^

The study was conducted using the LTD (Longitudinal Treatment Dynamics) database, which was established in 2012 and contains pseudonymized prescribed drug dispensing data collected from a panel of approximately 7000 community pharmacies in continental France. The pharmacies included in the panel (referred to hereafter as “panel pharmacies”) represent approximately 30% of all French community pharmacies and are representative in terms of geographical spread in continental France and covered population age.^17,18^ Only drugs dispensed in association with prescriptions for ambulatory care were considered in this study. This database contains information on patients (anonymized unique identifier, year of birth, and sex), dispensed drugs (name, unique identifier, EphMRA code [a drug identification code according to the anatomical classification system developed by the European Pharmaceutical Market Research Association^19^], dosage, volume, and pack form), dispensing events (prescribing and dispensing dates, the department of the dispensing pharmacy, and dispensed quantity), and prescribers (specialty and geographical location).

Drug dispensing data collection and processing within the LTD database were approved by the French Data Protection Authority on October 21, 2011; the approval was updated in July 2018 to comply with the European General Data Protection Regulation and applicable laws. Patient consent was not required because the data used in this study were fully deidentified. This study complies with the guidelines for Reporting of Studies Conducted Using Observational Routinely Collected Data for Pharmacoepidemiology (RECORD-PE).^20^

### Study Period

**Figure 1.**
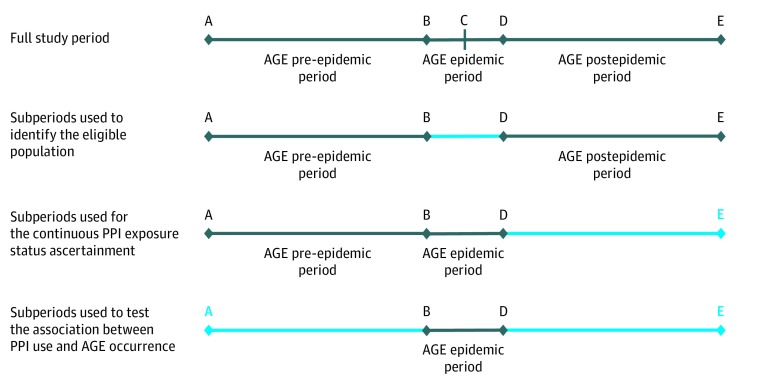
Description of the Study Period A winter season is composed of 3 subperiods defined as follows: an epidemic period is a 5-week period centered around the week with the highest acute gastroenteritis (AGE) incidence, a pre-epidemic period is the 100 days before the AGE epidemic period, and a postepidemic period is the 100 days after the AGE epidemic period. These periods were defined according to data collected by the French Sentinelles network. Points on the timelines are defined as follows: C refers to the week of AGE winter epidemic peak, B refers to point C minus 2 weeks; D refers to point C plus 2 weeks, A refers to point B minus 100 days, and E refers to point D plus 100 days. PPI indicates proton pump inhibitor.

The study was performed using data collected during the 2015 to 2016 winter season, which was cut into 3 subperiods (Figure 1): an epidemic period (ie, a 5-week period centered around the week with the highest AGE incidence), a pre-epidemic period (ie, the 100 days before the AGE epidemic period), and a postepidemic period (ie, the 100 days after the AGE epidemic period). These periods were defined on the basis of data collected by the Sentinelles network,^21,22^ a national surveillance network through which approximately 2% of French general practitioners voluntarily monitor several health indicators in primary care, including AGE. During the 2015 to 2016 winter season, the highest AGE incidence estimated from the Sentinelles data was observed in 2016 week 3, as defined by the International Organization for Standardization.^23,24^ Thus, the epidemic period lasted from 2016 week 1 to 2016 week 5 (ie, from January 4, 2016, to February 7, 2016); the pre-epidemic period lasted from September 26, 2015, to January 3, 2016; and the postepidemic period lasted from February 8, 2016, to May 17, 2016.

Previous studies^25,26,27^ have shown that AGE winter peaks are mainly caused by enteric viral pathogens. In the absence of microbiological tests to determine the cause of AGE, the AGE epidemic period was, therefore, chosen as the period during which to investigate the association between PPI therapy and viral AGE occurrence.

Data collected during the pre-epidemic and postepidemic periods were used to identify the population eligible for the study. In addition, data collected during the pre-epidemic period served as historical data for the assessment of the PPI exposure status and patient baseline characteristics; this period was considered long enough to capture dispensing events covering up to 90 days of treatment (the maximum dispensed volume allowed in France). The data analysis was conducted between January 2017 and December 2018.

### Study Population

The population eligible for the study included all patients with at least 1 drug dispensing event recorded during each of the pre- and postepidemic periods, documented year of birth and sex, and an identifiable regular panel pharmacy (ie, predominantly frequented by the patient during the study period) situated in continental France. These conditions were imposed to reduce the risk of including patients who died or migrated to a nonpanel pharmacy before or during the epidemic period.

### Definition of the Exposure

To ascertain exposure status, all PPI dispensing events collected during the pre-epidemic and epidemic periods were extracted from the database. All PPIs available in France were considered, including omeprazole, pantoprazole, lansoprazole, rabeprazole, and esomeprazole.

**Figure 2.**
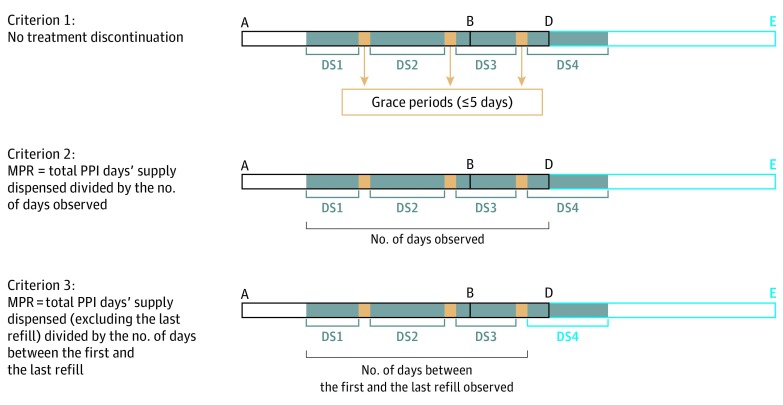
Example of Adherence and Persistence Criteria Evaluation for a Patient Exposed to Continuous Proton Pump Inhibitor (PPI) Treatment All PPI dispensing events collected during the pre-epidemic and epidemic periods (segments A to B and B to D, respectively) were extracted from the database and analyzed (segment D to E represents the postepidemic period). Assuming that 4 PPI dispensing events have been observed for this patient during these periods, the PPI days’ supply (DS; DS1, DS2, DS3, and DS4; the blue areas) was estimated for each of these dispensing events. A 5-day grace period between 2 successive dispensing events (yellow areas) was allowed before the treatment was considered interrupted. Criterion 1 is the persistence criterion in which there was no PPI therapy discontinuation (ie, all yellow areas are ≤5 days). Criterion 2 is the adherence criterion in which the medication possession ratio (MPR) is greater than or equal to 1, where MPR = (DS1 + DS2 + DS3 + DS4) / the number of days observed. The number of days observed is the number of days between the first PPI dispensing event observed and the end of the epidemic period. Criterion 3 is the adherence criterion in which MPR is greater than or equal to 1, where MPR = (DS1 + DS2 + DS3) / the number of days between the first and the last refill observed.

Proton pump inhibitor therapy was classified as continuous if 3 strict criteria were simultaneously fulfilled^28^: a persistence criterion implying no PPI therapy discontinuation (ie, the delay between any 2 successive dispensing events did not exceed the days’ supply estimated for the first dispensing event plus a grace period of 5 days), an adherence criterion based on the medication possession ratio (estimated as the total PPI days’ supply dispensed divided by the number of days observed), and another adherence criterion based on the medication possession ratio (estimated as the total PPI days’ supply dispensed, excluding the last refill, divided by the number of days between the first and the last refill). The PPI days’ supply dispensed was estimated as the dispensed quantity divided by the daily dose recommended in the drug monograph for the main indication for an adult. Each adherence criterion was fulfilled if the corresponding medication possession ratio was greater than or equal to 1. Figure 2 presents an example of how each criterion was evaluated.

Exposed patients were those receiving continuous PPI therapy initiated at least 30 days before the epidemic period (hereafter referred to as “continuous PPI users”). Patients receiving PPI therapy who did not fulfill at least 1 of these criteria were excluded from the study. Unexposed patients were those with no PPI dispensing events recorded in the database during the pre-epidemic and epidemic periods (hereafter referred to as “non–PPI users”).

### Definition of the Outcome

The outcome of interest was the occurrence of at least 1 AGE episode during the epidemic period. It was coded as a binary variable (≥ 1 AGE case vs none). The detection of AGE episodes from prescribed drug dispensing data was done by using an algorithm that identifies drug dispensing events corresponding to AGE treatments, taking into account patient characteristics, the delay between the prescription and drug dispensing event, and the type, number, and quantity of drugs dispensed.^29,30^ The types of drugs potentially prescribed to treat an AGE episode included oral rehydration salts, antiemetics, probiotic antidiarrheals, intestinal antipropulsives, intestinal absorbents, intestinal anti-infectious agents, and antispasmodic agents. Coprescriptions of these drugs and antibiotics, antineoplastic agents used in cancer therapy, gastric antacids, drugs used for inflammatory bowel diseases, or antiemetics in injectable form were considered to treat a disease other than AGE. This algorithm and the considered drug lists have been fully described and validated in a previous study^30^ that showed a strong overall agreement between the weekly AGE incidence estimated from the LTD drug dispensing database and the weekly AGE incidence estimated using clinical data collected at the Sentinelles network during winter seasons (Pearson correlation coefficient *r* ≥ 0.84). The agreement was strong in all age subgroups; the highest correlation was observed among adults aged 15 to 64 years (*r* ≥ 0.87) and the lowest among elderly patients (*r* ≥ 0.75).^30^

Patients whose AGE status could not be determined were excluded from the study. To limit the misclassification of long-term treatments as indicating AGE episodes, patients for whom the algorithm detected more than 4 AGE episodes during the pre-epidemic and epidemic periods were also excluded from the study.

### Confounding Factors

The confounding factors considered were age, sex, and treatments for the most common chronic conditions, which were identified on the basis of disease-specific anatomical therapeutic chemical classes: diabetes (class A10), cardiovascular diseases (classes B01, C01A, C01B, C01DA, C01DX, C01E, C02, C03, C07, C08, C09, and C10), obstructive airway diseases (class R03), and conditions requiring a psychotropic treatment (class N05 and N06). The presence of these conditions was inferred if disease-specific drugs had been dispensed at least once during the pre-epidemic or epidemic period.

### Validation and Sensitivity Analysis

The study was replicated for the 2016 to 2017 AGE winter season to check the reproducibility of results. Three sensitivity analyses were conducted. First, to investigate the presence of a potential selection bias related to past PPI use, the analyses were performed using a restricted population among exposed and unexposed patients: recent PPI users (ie, no PPI dispensing events recorded in the database earlier than 180 days before the pre-epidemic period) and never PPI users (ie, with no PPI dispensing events ever recorded in the database), respectively. Second, the analyses were performed using a relaxed persistence criterion: the time lag between 2 subsequent PPI dispensing events should not exceed twice the days’ supply estimated for the first dispensing event plus a grace period of 5 days. This criterion accounts for the winter holidays, which may affect an individual’s drug refill habits. Third, an analysis evaluating the association between AGE and histamine 2 receptor antagonists, another acid-suppression medication less potent than PPI, was performed using the same methods.

### Statistical Analysis

Non–PPI users were frequency matched to the continuous PPI users by groups of exact year of birth, sex, and identifiable regular panel pharmacy, using random sampling with no replacement for a ratio of 3 non–PPI users to 1 continuous PPI user. When 3:1 matches were not available, all available matches for the group were included.

To limit the risk of including patients with erroneous records, those with extreme values of the number of prescriptions or dispensing events were excluded from the analysis. Extreme values were defined as those over the third quartile plus thrice the interquartile range.^31^

Log-binomial regression models were used to estimate relative risks (RRs) and 95% CIs for the overall study population and for the age groups 0 to 14 years, 15 to 44 years, 45 to 64 years, 65 to 74 years, and 75 years or older. To assess whether the association between continuous PPI therapy and the occurrence of AGE during winter epidemic periods varied according to age, formal tests for interaction between exposure status and age group were conducted, using the analysis of deviance. The number needed to harm was estimated as the inverse of the absolute risk increase.^32^

All analyses were performed using R Studio statistical software version 1.2.1335 (R Project for Statistical Computing),^33^ with packages sqldf, data.table, dplyr, and glm. Statistical significance was defined as *P* < .05; all alternate hypotheses were 2-sided.

## Results

**Figure 3.**
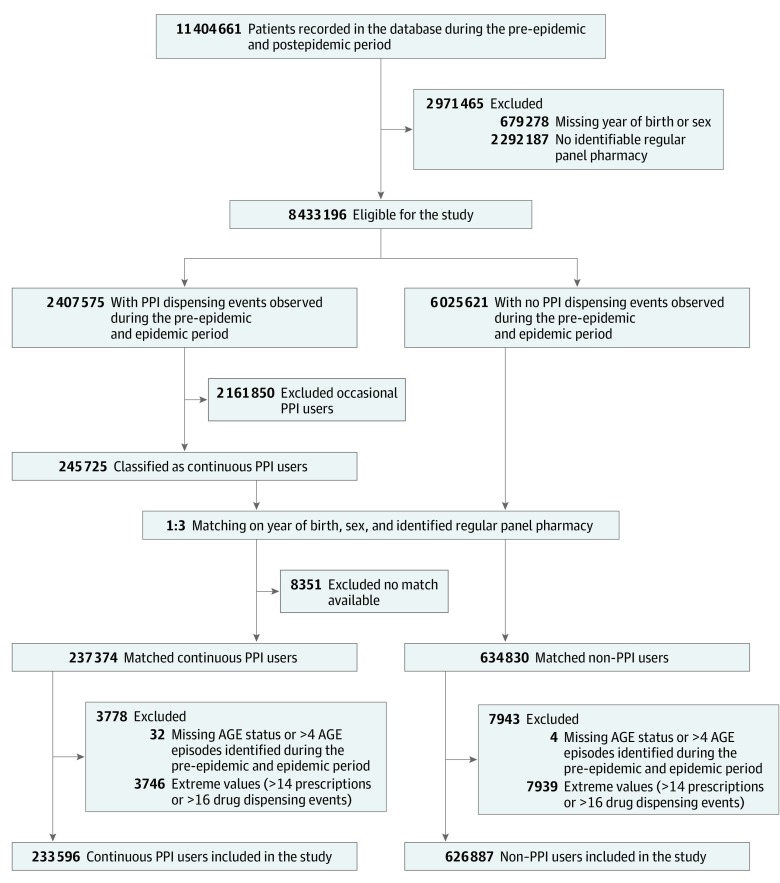
Selection of Patients Included in the Study Flowchart shows inclusion and exclusion criteria for this study. AGE indicates acute gastroenteritis; and PPI, proton pump inhibitor.

There were 233 596 continuous PPI users and 626 887 non–PPI users included in the study, after applying inclusion and exclusion criteria (Figure 3). Among the patients eligible for the study, 28.5% had at least 1 PPI dispensing event, and 2.9% were receiving continuous PPI therapy. The baseline characteristics of the included patients are summarized in Table 1. The median (interquartile range) age was 70 (61-80) years in the non–PPI users group (56.3% female) and 71 (62-81) years in the continuous PPI users group (55.8% female). Compared with non–PPI users, continuous PPI users were dispensed more treatments for cardiovascular diseases (80.6% vs 69.1%), diabetes (19.6% vs 14.7%), psychiatric diseases (50.0% vs 31.5%), and obstructive airway diseases (20.1% vs 11.4%).

**Table 1.** Baseline Characteristics of the Population Included in the Study

Characteristic	Exposure Group, No. (%)	
	Non–PPI Users (n = 626 887)	Continuous PPI Users (n = 233 596)
Age, median (interquartile range), y	70 (61-80)	71 (62-81)
Age group, y		
0-4	659 (0.1)	239 (0.1)
5-14	338 (0.1)	141 (0.1)
15-44	25 648 (4.1)	9788 (4.2)
45-64	181 536 (29.0)	63 984 (27.4)
65-74	182 984 (29.2)	64 721 (27.7)
≥75	235 722 (37.6)	94 723 (40.5)
Sex		
Female	353 128 (56.3)	130 285 (55.8)
Male	273 759 (43.7)	103 311 (44.2)
Treatments for chronic conditions		
Cardiovascular diseases	433 357 (69.1)	188 379 (80.6)
Diabetes	92 335 (14.7)	45 788 (19.6)
Psychiatric diseases	197 478 (31.5)	116 873 (50.0)
Obstructive airway diseases	71 574 (11.4)	46 946 (20.1)

Abbreviation: PPI, proton pump inhibitor.

At least 1 AGE episode was observed during the epidemic period for 3131 continuous PPI users (1.3%) compared with 4327 non–PPI users (0.7%). All RRs estimated from the primary analysis and PPI sensitivity analyses are reported in Table 2. After adjusting for confounding factors, the risk of AGE was significantly higher among continuous PPI users compared with non–PPI users, all age groups considered (adjusted RR [aRR], 1.81; 95% CI, 1.72-1.90) and among age groups 45 to 64 years (aRR, 1.66; 95% CI, 1.54-1.80), 65 to 74 years (aRR, 2.19; 95% CI, 1.98-2.42), and 75 years and older (aRR, 1.98; 95% CI, 1.82-2.15). No significant association was observed in age groups 0 to 14 years and 15 to 44 years. Those age groups represented less than 5% of the exposed population identified in the database. A significant interaction between exposure status and age was observed (*P* for interaction < .01). The estimated number needed to harm was 153, all ages considered. The significantly increased risk of AGE associated with continuous PPI therapy was consistent across validation and sensitivity analyses, with aRRs ranging from 1.68 (95% CI, 1.44-1.94) to 1.89 (95% CI, 1.82-1.97) overall, from 1.63 (95% CI, 1.25-2.14) to 1.80 (95% CI, 1.69-1.92) among those aged 45 to 64 years, from 1.90 (95% CI, 1.73-2.09) to 2.24 (95% CI, 1.60-3.14) among those aged 65 to 74 years, and from 1.80 (95% CI, 1.67-1.94) to 1.99 (95% CI, 1.86-2.13) among those aged 75 years and older.

**Table 2.** RR of AGE in Patients Exposed to Continuous PPI Therapy Compared With Unexposed Patients

Analysis	Patients With AGE, No./Total (%)		RR (95% CI)	
	Continuous PPI Users	Non–PPI Users	Crude	Adjusted^a^
Main analysis^b^				
All ages	3131/233 596 (1.3)	4327/626 887 (0.7)	1.94 (1.86-2.03)	1.81 (1.72-1.90)
Age groups, y				
0-14	23/380 (6.1)	52/997 (5.2)	1.16 (0.72-1.87)	1.07 (0.66-1.75)
15-44	224/9788 (2.3)	489/25 648 (1.9)	1.20 (1.03-1.40)	1.18 (1.00-1.39)
45-64	1030/63 984 (1.6)	1622/181 536 (0.9)	1.80 (1.67-1.95)	1.66 (1.54-1.80)
65-74	771/64 721 (1.2)	910/182 984 (0.5)	2.40 (2.18-2.64)	2.19 (1.98-2.42)
≥75	1083/94 723 (1.1)	1254/235 722 (0.5)	2.15 (1.98-2.33)	1.98 (1.82-2.15)
Reproducibility and sensitivity analyses for 2016 to 2017 AGE winter season^b^				
All ages	3630/244 782 (1.5)	5308/656 348 (0.8)	1.83 (1.76-1.91)	1.74 (1.66-1.81)
Age groups, y				
0-14	27/419 (6.4)	70/1104 (6.3)	1.02 (0.66-1.56)	1.08 (0.70-1.66)
15-44	295/10 325 (2.9)	588/27 019 (2.2)	1.31 (1.14-1.51)	1.33 (1.15-1.53)
45-64	1230/66 286 (1.9)	1929/187 949 (1.0)	1.81 (1.68-1.94)	1.73 (1.61-1.87)
65-74	826/69 379 (1.2)	1112/196 527 (0.6)	2.10 (1.92-2.30)	1.90 (1.73-2.09)
≥75	1252/98 373 (1.3)	1609/243 749 (0.7)	1.93 (1.79-2.08)	1.80 (1.67-1.94)
Recent PPI users vs never PPI users^b^				
All ages	332/26 540 (1.3)	421/59 733 (0.7)	1.77 (1.54-2.05)	1.68 (1.44-1.94)
Age groups, y				
0-14	22/282 (7.8)	42/740 (5.7)	1.37 (0.84-2.26)	1.26 (0.76-2.08)
15-44	31/1581 (2.0)	63/3262 (1.9)	1.01 (0.66-1.55)	1.13 (0.73-1.75)
45-64	98/7458 (1.3)	131/16 905 (0.8)	1.70 (1.31-2.20)	1.63 (1.25-2.14)
65-74	75/6608 (1.1)	71/15 704 (0.5)	2.51 (1.82-3.47)	2.24 (1.60-3.14)
≥75	106/10 611 (1.0)	114/23 122 (0.5)	2.03 (1.56-2.64)	1.85 (1.41-2.42
Relaxed persistence criteria^b^				
All ages	4850/351 761 (1.4)	6275/919 486 (0.7)	2.02 (1.95-2.10)	1.89 (1.82-1.97)
Age groups, y				
0-14	36/561 (6.4)	69/1482 (4.7)	1.38 (0.93-2.04)	1.33 (0.89-1.97)
15-44	377/14 818 (2.5)	702/38 650 (1.8)	1.40 (1.24-1.59)	1.38 (1.21-1.57)
45-64	1620/96 375 (1.7)	2346/270 332 (0.9)	1.94 (1.82-2.06)	1.80 (1.69-1.92)
65-74	1180/97 210 (1.2)	1355/269 319 (0.5)	2.41 (2.23-2.61)	2.20 (2.03-2.39)
≥75	1637/142 797 (1.2)	1803/339 703 (0.5)	2.16 (2.02-2.31)	1.99 (1.86-2.13)

Abbreviations: AGE, acute gastroenteritis; PPI, proton pump inhibitor; RR, relative risk.

^a^ Multivariable log-binomial model; in the all-ages analyses, the model was adjusted for age, sex, and treatments for diabetes, cardiovascular diseases, obstructive airway diseases, and conditions requiring a psychotropic treatment. Models were adjusted for age and sex to account for remaining imbalances due to the uneven number of matches per exposed patient.

^b^ For all analyses, *P* < .01 for interaction between age group and receipt of continuous PPI therapy.

There were 1681 patients exposed to continuous histamine 2 receptor antagonists therapy identified in the database, who were matched to 4739 unexposed patients. A significant overall association was found between the continuous use of histamine 2 receptor antagonists and AGE occurrence (aRR, 2.08; 95% CI, 1.27-3.39). Samples sizes were too small for age subgroup analyses.

## Discussion

In this study based on a large nationwide drug dispensing database, the exposure to continuous PPI therapy was associated with an increased risk of AGE occurring during periods of circulation of enteric viruses. Under the assumption of a causal association, 1 additional patient could have AGE during winter epidemic periods for 153 patients receiving continuous PPI therapy.

To our knowledge, this is the first study investigating the association between continuous PPI use and the occurrence of AGE during winter epidemics of viral AGE in ambulatory care. The main strength of this study is the use of a nationwide database of pharmacies, capturing approximately 30% of the French population. Among the patients eligible for the study from this database, 28.5% had at least 1 PPI dispensing event, and 2.9% were receiving continuous PPI therapy. These figures are consistent with administrative data on PPI use in the whole French population.^34^

Most studies investigating the association between PPI exposure and infectious gastroenteritis have focused on adult populations, which are the most exposed to PPI. Less is known about this association in the youngest populations, for whom the highest AGE incidence is usually observed.^27^ The current study was performed in the overall population, including children and young adults. No association between continuous PPI use and AGE occurrence was observed in the main analysis for patients younger than 45 years. However, continuous PPI use is less common in these age groups. They represented less than 5% of the exposed population identified in the database. Thus, the results regarding these subgroups should be interpreted with caution.

Another strength of this study is the use of a strict definition of exposure, based on 3 restrictive adherence and persistence criteria. Because PPI treatment coverage was estimated according to the dose recommended in the drug monograph for the main indication for an adult, misclassification of exposure status may still occur, particularly among children, for whom dosage depends on age and weight. However, such misclassification of exposure, if any, would tend toward classifying actual continuous PPI users as occasional PPI users and excluding them from the study, rather than the opposite, which would bias toward the null.

### Interpretation of Results and Comparison With Previous Studies

Results reported in this study are in line with those reported in a Swedish study^10^ investigating the association between PPI use and norovirus infection in hospitalized adults (odds ratio, 1.73; 95% CI, 1.07-2.81). Compared with the Swedish study, the current study was performed using community data and relied on a large sample, including young children.

Formal tests for interaction showed that the studied association varied with the patient’s age. Patients aged 45 years and older receiving continuous PPI therapy had a significantly higher risk of developing AGE during periods of circulation of enteric viruses compared with those not using PPI therapy. No significant association was observed among younger patients. The pathogens causing viral enteric infections are different according to age groups: the norovirus is predominantly responsible for winter AGE in adult populations, but children are mostly infected with the rotavirus.^25,35^ This could contribute to the different results observed across age groups.

Although the pathophysiological association between PPI use and viral enteric infections is still unclear, it has been shown that PPI use induces changes in the gut microbiome and that microbiota changes can affect enteric virus pathogenesis, suggesting a possible biological association.^11,36^ A direct effect of gastric acid suppression on the risk of enteric viral infections seems plausible, because the same association was observed between histamine 2 receptor antagonists and AGE.

### Research and Clinical Perspectives

Observational studies based on nationwide databases provide valuable estimates of the association between continuous PPI therapy and potential adverse events. More studies in different settings are needed to confirm the association reported here. Investigating the dose-response relationship and the pathophysiological mechanisms of the interaction between PPI use and the risk of contracting enteric viruses may help infer a causal association.

One-half of continuous PPI prescriptions are inappropriate.^37^ Associations such as those reported in this study are yet another reason to reexamine unnecessary and non–evidence-based indications for PPI therapy. As regions head into enteric viral infection season, this would be an opportune time to reassess the ongoing need for PPI therapy in patients, especially older adults, and potentially deprescribe.

### Limitations

The study has several limitations, mainly related to PPI exposure and AGE status ascertainment. First, diagnoses were not available, and ascertainment of the AGE status relied solely on drug dispensing data. The drugs used to treat AGE are not disease specific, and treatment indication depends on patient characteristics. To overcome this limitation, AGE episodes were identified on the basis of a previously validated algorithm.^29,30^ In addition, to reduce the risk of misclassifying other chronic diseases as AGE episodes, patients for whom the algorithm detected more than 4 AGE episodes were excluded from the analysis.

Second, the actual PPI doses were unavailable, and a dose-response relationship could not be investigated. Third, there is a risk of incomplete follow-up, which may result in misclassification of outcome or exposure status. This may occur if patients alternate between panel and nonpanel pharmacies, if PPI or AGE drugs are purchased over the counter (which is possible in France), or if patients receive non–ambulatory care prescriptions. However, to be included in the study, patients were required to have had at least 1 drug dispensing event in the pre-epidemic period and 1 in the postepidemic period and an identifiable regular panel pharmacy.

Fourth, the use of a strict exposure definition may have resulted in the exclusion of a population receiving continuous PPI therapy that did not conform to the criteria considered in this study. The sensitivity analysis based on a relaxed persistence definition yielded results similar to the main analysis, suggesting that such selection bias, if any, was limited.

Fifth, information on additional potential confounders or effect modifiers (eg, sociodemographic characteristics, food consumption, potentially contagious contacts, body mass index, smoking, and alcohol consumption) were not available. Only the comorbidities whose treatment rely on disease-specific drug classes could be measured and accounted for. They were indeed more frequent in PPI users. These comorbidities could result in more-frequent contact with the health care system and, thus, a greater likelihood of receiving a diagnosis of and treatment for AGE; patients who are not as ill may not seek medical care for viral gastroenteritis. Thus, the presence of comorbidities might be a confounder of the investigated association, on which the models were adjusted. Imbalances observed between exposed and unexposed patients for the measured confounders may also be present in other unmeasured confounding factors. However, the association estimated in this study was significant (particularly among elderly patients), and it remained relatively stable after adjustment for the measured confounders.

Sixth, even though patients with antibiotics prescribed along with AGE drugs were not counted as AGE cases, other antibiotic dispensing events were not taken into account. Seventh, confounding by indication cannot be ruled out, but we could not find any published evidence linking AGE risk and disorders for which PPIs are indicated (eg, gastroesophageal reflux or peptic ulcer disease).

## Conclusions

Continuous exposure to PPI therapy among individuals aged 45 years and older was associated with an increased risk of developing AGE during periods of highest circulation of enteric viruses. Despite the limitations related to exposure and outcome ascertainment and potential residual confounding, the results reported in this study support the hypothesis that continuous PPI use is associated with an increased risk of infections with enteric viruses and motivate the need for further studies to confirm this association and investigate the pathophysiological mechanisms.
